# Recent progress of methods for cuproptosis detection

**DOI:** 10.3389/fmolb.2024.1460987

**Published:** 2024-09-04

**Authors:** Ligang Zhang, Ruiting Deng, Raoqing Guo, Yawen Jiang, Yichen Guan, Caiyue Chen, Wudi Zhao, Guobin Huang, Lian Liu, Hongli Du, Dongsheng Tang

**Affiliations:** ^1^ Gene Editing Technology Center of Guangdong Province, School of Medicine, Foshan University, Foshan, China; ^2^ School of Biology and Biological Engineering, South China University of Technology, Guangzhou, China; ^3^ Beijing Mercer United International Education Consulting Co., Ltd., Guangzhou, China; ^4^ State Key Laboratory of Respiratory Disease, Guangzhou Institute of Respiratory Health, The First Affiliated Hospital of Guangzhou Medical University, Guangzhou, China

**Keywords:** cuproptosis, detection methods, cell death, Cu content, morphology, molecular biology, biochemical pathways

## Abstract

Varying from other identified cell death pathways, cuproptosis is a new type of regulated cell death characterized by excess Cu ions, abnormal aggregation of lipoylated proteins in TCA cycle, loss of Fe-S cluster proteins, upregulation of HSP70, leading to proteotoxic and oxidative stress. Cuproptosis is highly concerned by scientific community and as the field of cuproptosis further develops, remarkable progress has been made in the verification and mechanism of cuproptosis, and methods used to detect cuproptosis have been continuously improved. According to the characteristic changes of cuproptosis, techniques based on cell death verification, Cu content, morphology, molecular biology of protein levels of cuproptosis-related molecules and biochemical pathways of cuproptosis-related enzyme activity and metabolites of oxidative stress, lipoic acid, TCA cycle, Fe-S cluster proteins, oxidative phosphorylation, cell respiration intensity have been subject to cuproptosis verification and research. In order to further deepen the understanding of detecting cuproptosis, the principle and application of common cuproptosis detection methods are reviewed and categorized in cellular phenomena and molecular mechanism in terms of cell death, Cu content, morphology, molecular biology, biochemical pathways with a flow chart. All the indicating results have been displayed in response to the markers of cuproptosis, their advantages and limitations are summaried, and comparison of cuproptosis and ferroptosis detection is performed in this study. Our collection of methods for cuproptosis detection will provide a great basis for cuproptosis verification and research in the future.

## 1 Introduction

**FIGURE 1:**
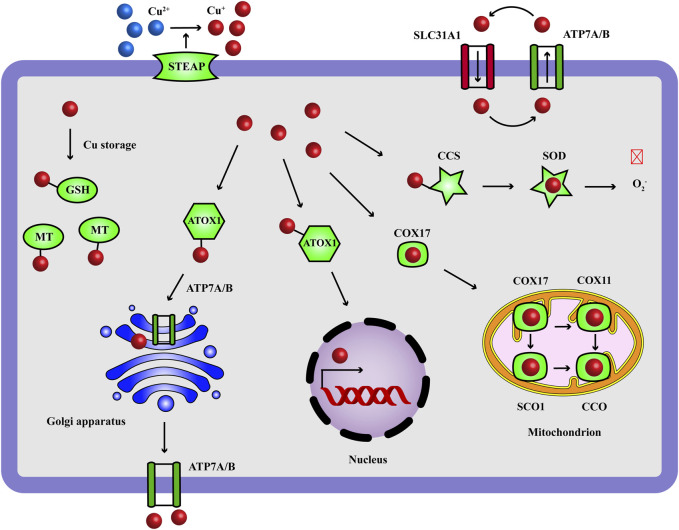
Copper homeostasis: the transport and functions of Cu ions. Cu ions are transported to target cells through blood circulation and the intracellular input and output of Cu^+^ (reduced by STEAP) are mediated by SLC31A1 and ATP7A/7B. GSH is a Cu chaperone and the intracellular Cu ions are stored with MT. Cu ions act as a catalytic cofactor to activate the target enzymes in oxidative balance (SOD), mitochondrial respiration (ETC.), transcriptional complex (nucleus) and protein processing (Golgi apparatus). CCS carries Cu^+^ to SOD, ATOX1 carries Cu^+^ to nucleus and Golgi apparatus, COX17 carries Cu^+^ to SCO1 and COX11 in mitochondrion for the assembly of CCO. Red and crossed symbol indicates inhibitory effect. ATOX1, antioxidant-1; CCO, cytochrome c oxidase; CCS, Cu chaperone for superoxide dismutase; COX, cytochrome c oxidase; GSH, glutathione; MT, metallothionein; SCO, synthesis of cytochrome c oxidase; SLC31A1, solute carrier family 31 member 1; SOD, superoxide dismutase; STEAP, metal reductase prostate six-transmembrane epithelial antigen.

Copper (Cu) is an essential micronutrient that acts as a vital catalytic cofactor to activate the target enzymes in a wide range of physiological process, including protein processing (Perkal et al., 2020), oxidative balance (Pan et al., 2024), mitochondrial respiration (Garza et al., 2023) and intranuclear transcriptional regulation (Zhong et al., 2023). Our body maintains the concentration of Cu ions at a very low level through some active internal environmental balancing mechanisms: the absorption of Cu ions occurs mainly in gastrointestinal tract, where Cu^2+^ are reduced to Cu^+^ by metal reductase prostate six-transmembrane epithelial antigen (STEAP), and reach to liver via the bloodstream; the uptake of Cu^+^ is mediated by solute carrier family 31 member 1 (SLC31A1) and Cu ions are stored by metallothionein (MT) in hepatocytes; Cu-atpase ATP7B in liver delivers Cu ions to the secretory pathway for ceruloplasmin metalation and Cu ions are carried to tissues and organs, in which ATP7A functions; homeostasis regulation of intracellular Cu content is influenced by a complex Cu-dependent network, including Cu enzymes, Cu chaperones and membrane transporters; they work together to mediate the input, output, and intracellular utilization of Cu ions, which maintain intracellular Cu levels within a specific range, and prevent the cell damage of Cu deficiency and overload (Figure 1) (Chen et al., 2022; Garza et al., 2023; Bartee and Lutsenko, 2007). More and more evidence shows that the imbalance of Cu homeostasis is related to the occurrence and development of adverse health effects, such as Menke’s disease (Fujisawa et al., 2022), Wilson’s disease (Stalke et al., 2023), neurodegenerative diseases (Alzheimer’s disease, amyotrophic lateral sclerosis, Huntington’s disease) (Shen et al., 2023; Yang Y. et al., 2023; Cordeiro et al., 2023), cardiovascular diseases (Yang L. et al., 2023) and cancers (Xie et al., 2023).

**FIGURE 2:**
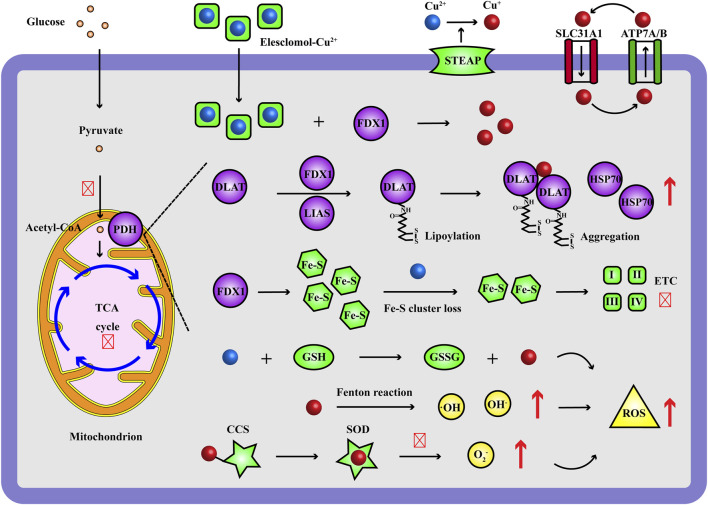
Occurrence and mechanism of Cu ions inducing cell death. During cuproptosis, the Elesclomol-delivered Cu^2+^ target FDX1 and are reduced to Cu^+^, which bind to the lipoylated DLAT of PDH complex in mitochondrial TCA cycle and induce aggregation of DLAT, up-regulation of HSP70, loss of Fe-S cluster proteins, ETC disruption; moreover, excess Cu ions improve Fenton reaction, impair GSH and SOD antioxidant system, resulting in increasing ROS levels (·OH, OH^−^, O_2_^−^). Red and up arrow indicates increasing effect, red and crossed symbol indicates inhibitory effect. CCS, Cu chaperone for superoxide dismutase; DLAT, dihydrolipoamide S-acetyltransferase; ETC, electron transport chain; FDX1, ferredoxin 1; GSH, glutathione; GSSG, glutathione; HSP70, heat shock protein 70; LIAS, lipoyl synthase; PDH, pyruvate dehydrogenase; ROS, reactive oxygen species; SLC31A1, solute carrier family 31 member 1; SOD, superoxide dismutase; STEAP, metal reductase prostate six-transmembrane epithelial antigen; TCA, tricarboxylic acid.

Recently, Golub TR et al. reported a novel form of cell death caused by excess Cu ions, named cuproptosis (Tsvetkov et al., 2022). Cuproptosis mainly shows a series of characteristic changes: the excess Cu ions delivered by Cu ionophores (Elesclomol, Disulfiram, etc.) directly bind to the ferredoxin 1 (FDX1)/lipoyl synthase (LIAS)-lipoylated target dihydrolipoamide S-acetyltransferase (DLAT) in mitochondrial tricarboxylic acid (TCA) cycle and induce abnormal aggregation of DLAT, loss of Fe-S cluster proteins and upregulation of heat shock protein 70 (HSP70), leading to proteotoxic stress, oxidative stress and eventually cell death (Figure 2). Cuproptosis is a new type of regulated cell death (RCD) impairing mitochondrial respiration and highly concerned by scientific community, varying from other identified programmed cell death such as apoptosis (Ren Y. et al., 2023), necrosis (Sui et al., 2023), pyroptosis (Liu et al., 2022), autophagy (Tao et al., 2023) and ferroptosis (Wang et al., 2023b). Specifically, from the discovery of cuproptosis, 10 genes have been verified as cuproptosis-related genes, including positive regulatory factors: FDX1, lipoylation proteins [lipoic acid pathway lipoacyltransferase 1 (LIPT1), LIAS, dihydroacylamide dehydrogenase (DLD)], lipoylated targets [DLAT, pyruvate dehydrogenase E1 subunit α 1 (PDHA1), pyruvate dehydrogenase E1 subunit β (PDHB)] and negative regulatory factors: metal regulatory transcription factor 1 (MTF1), cyclin-dependent kinase inhibitor 2A (CDKN2A), glutaminase (GLS) (Tsvetkov et al., 2022).

According to these characteristic changes of cuproptosis, detection methods involving in cell death verification, Cu content, morphology, molecular biology of protein levels of cuproptosis-related molecules and biochemical pathways of cuproptosis-related enzyme activity and metabolites of oxidative stress, lipoic acid, TCA cycle, Fe-S cluster proteins, oxidative phosphorylation, cell respiration intensity, have been widely applied in exploring the verification and molecular mechanism of cuproptosis. For further enhancing the understanding of cuproptosis detection methods, we categorize the principle and application of common cuproptosis detection methods with a flow chart, summarize their advantages and limitations, and perform comparison of cuproptosis and ferroptosis detection in this study, hoping to provide a great basis for verifying and studying cuproptosis in the future.

## 2 Detection based on cell death

The Cu homeostasis imbalance will induce Cu toxicity to cells through aggravating Cu import and reducing Cu efflux. And it has been demonstrated that Cu ionophores (Elesclomol, Disulfiram, etc.) deliver excess Cu^2+^ into cells and result in a new type of RCD, which can be rescued by pretreatment of Cu chelators (Tsvetkov et al., 2022). Firstly, we should identify the induction of cell death effects which may be mediated by excess Cu ions. Assays of CCK8/MTT (indicated by absorbance), clone formation, Annexin V/PI (flow cytometry) and xenograft (*in vivo* test) are common methods to verify cell viability and cell death effects in Cu stress, with the results of lower optical density (OD), less cell quantity, higher apoptosis rate and smaller xenograft, respectively. Since cell death pathways are very complex, cell death inhibitors based on sensitivity are recognized regents to exclude classic cell death pathways during cuproptosis study, involving in apoptosis [Z-VAD-FMK (Yan et al., 2021), Z-DEVD-FMK (Deng et al., 2021)], necrosis [Necrostatin-1 (Sun et al., 2023), Necrosulfonamide (Gao et al., 2023)], pyroptosis [BAY 11-7082 (Wu et al., 2021), Ac-YVAD-cmk (Lu et al., 2024)], autophagy [Bafilomycin A1 (Zhang et al., 2023), MHY1485 (Lin et al., 2020)] and ferroptosis [Ferrostatin-1 (Yu et al., 2022), Cycloheximide (Zheng et al., 2023)]. To preliminarily identify the cell death triggered by Cu ions, inhibitory effects on cell viability should be measured, which are not sensitive to inhibitors of classic cell death pathways but pretreatment of Cu chelators.

## 3 Detection based on Cu content

Cu ions should be kept in a narrow range for normal cellular metabolic activities. After the preliminary verification of cell death effects in Cu stress, next, it’s essential to measure the intracellular Cu ion levels, a key procedure used to cuproptosis. Researchers found that Elesclomol as low as 40 nM increased tenfold intracellular Cu ion levels (indicated by inductively coupled plasma mass spectrometry, ICP-MS) and triggered cell death after 24 h, but the inhibitory effects could be reversed when pretreating with Cu chelators or in the absence of Cu ions, indicating that the cell death was induced by the accumulation of Cu ions, not the Cu ionophore itself (Tsvetkov et al., 2022). Herein, the intracellular Cu ion levels should be significantly increased during cuproptosis so that the Cu content awaits measurement when the cell death effects have been observed in Cu stress. ICP-MS is one of the most accurate and sensitive technologies for quantifying metal ion content, but limited by complex testing preparation and instrument. Thus, some other effective methods are required to accurately and quickly measure Cu content in serum and cell lysate, including colorimetric, fluorescent probe, and electrochemistry methods (Table 1).

**TABLE 1:** Common methods to detect Cu content.

Assays	Principle	Sensitivity (μM)	Advantages	Disadvantages	Ref.
MesGen #MCK4575	Colorimetric	1.1–47.21	Sensitive Accurate High-throughput	Interfered by metal chelators Microplate reader needed Store at 4°C	Tong et al. (2023)
Elabscience #E-BC-K775-M	Colorimetric	0.18–5			Li et al. (2023)
BioAssay Systems #DICU-250	Colorimetric	1.0–47			Parmar et al. (2018)
Au nanoclusters (AuNCs)	Fluorescent probe	0.5–10	More sensitive Accurate High-throughput	Interfered by other metal ions Fluorescent reader needed	Ou et al. (2018)
Quantum dots (QDs)	Fluorescent probe	0.1–10		Toxicity Fluorescent reader needed	Liu and Sun (2023)
Ti_3_C_2_T_x_/MWNTs-Au	Electrochemistry	0.16–7.87	Very sensitive Accurate High-throughput	High background signal Results unvisualized Electrochemical workstation needed	Hui et al. (2020)
CdS QDs/WO_3_ nanoflakes	Photo electrochemistry	0.01–0.5	Low background signal Results visualized High portability	Electrochemical workstation needed	Zhang et al. (2022a)

### 3.1 Colorimetric

**FIGURE 3:**
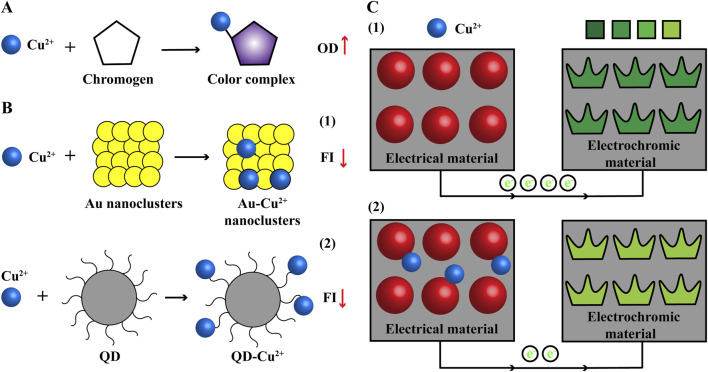
Common methods used to detect Cu content **(A)** Colorimetric. Cu ions and chromogen form color complex with increasing OD. **(B)** Fluorescent probe. The FI of noble metallic nanoclusters (Au) and QD will be quenched when combined with Cu ions. **(C)** Electrochemistry. The electrical signal of electrical material will be changed and recorded in the presence of Cu ions and signal can be visual with combination of electrochromic material. e^−^: electron; FI, fluorescence intensity; OD, optical density; QD, quantum dot.

Chromogen specifically forms color complex with Cu ions and the intensity proportional to Cu content can be measured using a microplate reader, such as copper assay kits from MesGen (Tong et al., 2023), Elabscience (Li et al., 2023) and BioAssay Systems (Parmar et al., 2018). They are simple, sensitive, accurate and high-throughput, but will be interfered by metal chelators in samples, and microplate reader needed (Figure 3A).

### 3.2 Fluorescent probe

Fluorescent probe is a kind of fluorescence signal nanomaterial with high sensitivity and short reaction time, which is widely used in chemical biological sensing, biomedical imaging and so on. The fluorescence signal of noble metallic nanoclusters and quantum dots will be quenched in the presence of Cu ions (Figure 3B). Thus, they have been developed to detect Cu content, with advantages of more sensitive, accurate and high-throughput, but fluorescent reader needed (Ou et al., 2018; Liu and Sun, 2023).

### 3.3 Electrochemistry

Scientists use the highly electrical material (Ti_3_C_2_T_x_/MWNTs-Au) as the working electrode and the electrical signal will be changed and recorded when binding to Cu ions (Hui et al., 2020). But electrochemistry technique has high background signal and the results are unvisualized. The electrochromic material has stable and reversed color change in electric field. Further, the electrochemistry technique and electrochromic material are combined that electrochromic material is used as the counter electrode (CdS QDs/WO_3_ nanoflakes); Cu ions bind to the electrical material and reduce the electrical signal, which causes the color change of electrochromic material from dark to light. Visual signal of Cu ions can be obtained from color change and used for fast semiquantitative field detection; electrical signal can provide more accurate data for Cu content (Figure 3C) (Zhang N. et al., 2022). Electrochemical workstation is needed for this method of detecting Cu content.

## 4 Detection based on morphology

In the first step and second step, the cell death effects rescued by pretreatment of Cu chelators and Cu deposition can indicate the induction of cuproptosis. Since some specific morphological properties will appear during cuproptosis, morphological detection can intuitively distinguish cuproptosis. Then we can check the morphology properties of cell membrane and organelles for adjuvant estimation. According to the characteristics of cuproptosis, Yang L. et al. summarized that the morphological features of cuproptosis involve cell membrane rupture, mitochondrial contraction, endoplasmic reticulum and chromatin damage (Yang et al., 2023a). These morphological changes of subcellular structure and organelles can be intuitively examined by transmission electron microscope (TEM). Morphology method has intuitive and clear results, but the sample preparation is complicated and there are subjective biases in the observation of subcellular morphology, so it is often combined with quantitative detection methods based on cell death and Cu content we describe above to preliminarily verify cuproptosis. By far methods based on cell death effects with inhibitors, Cu ions overload and morphological features may thus be promising tools for detecting the cellular phenomena of cuproptosis, further investigation about molecular mechanism is needed to gain comprehensive evaluation of cuproptosis. Therefore, addressing the detection methods of Cu-induced cell death based on molecular biology of protein levels of cuproptosis-related molecules, biochemical pathways of cuproptosis-related enzyme activity and metabolites presents a challenging and inevitable undertaking.

## 5 Detection based on molecular biology

According to the discovery of cuproptosis, it is proved to be related to the collapse of Cu homeostasis and mitochondrial respiration mediated by Cu ionophores, and the specific changes of protein levels of cuproptosis-related molecules including lipoylation process, electron transport chain (ETC), HSP70 are involved in this new type of cell death (Figure 2). Beyond that, abnormal gene expression of Cu transport and Cu chaperones may also lead to the Cu homeostasis disruption and more seriously the progress of genetic, neurodegenerative, cardiovascular, tumoral diseases (Chen et al., 2022; Tsvetkov et al., 2022). Evaluating these molecular biology of protein levels of cuproptosis-related molecules are essential to quantitatively verify the occurrence of cuproptosis, which include Cu ionophore-Cu-induced cell death (5.1–5.3) and endogenous Cu homeostasis imbalance (5.4–5.5) in this section.

### 5.1 Lipoylation: FDX1, DLAT

Scientists proved that mitochondrial respiration was required for cuproptosis and metabolite analysis showed a time-dependent increase in the dysregulation of TCA cycle-related metabolites in cuproptosis cells; genome-wide CRISPR/Cas9 function loss screening identified relevant genes reducing cuproptosis: FDX1, lipoylation proteins (LIPT1; LIAS; DLD) and lipoylated targets (PDH complex: DLAT, PDHA1, PDHB) (Tsvetkov et al., 2022; Solmonson and DeBerardinis, 2018). Thus the cells with high levels of FDX1 and DLAT are more sensitive to Cu cell death effects. FDX1 encodes a reductase that reduces Cu^2+^ to more toxic form Cu^+^ and its content in cells and tissues can be determined using real-time quantitative PCR (qRT-PCR), western blot, immunohistochemistry (IHC), ELISA assays (Zulkifli et al., 2023). In addition, FDX1 was proved to be an upstream regulator of protein lipoylation and regulated the lipoylation of DLAT cooperated with LIAS, essential for PDH complex and TCA cycle (Tsvetkov et al., 2022). qRT-PCR, western blot, IHC, immunoprecipitation, mass spectrometry assays can be performed to measure the expression and lipoylation (indicated by DLAT antibody and lipoic acid antibody) of DLAT (Nowinski et al., 2020). Cu can bind free lipoic acid with a considerable affinity so that the lipoyl moiety of DLAT is required for Cu binding.

One of the markers of cuproptosis is triggering the aggregation of lipoylated target DLAT due to the binding to Cu^+^, leading to the reduction of free monomer of DLAT (Tsvetkov et al., 2022). The aggregation of Cu^+^-DLAT can be captured by immunofluorescence (IF), nondenaturing gel electrophoresis/western blot, high performance liquid chromatography (HPLC), and alternative methods include dynamic light scattering (DLS), Taylor dispersion analysis (TDA), electrospray differential mobility analysis (ES-DMA) (Wang and Roberts, 2018).

### 5.2 Fe-S cluster proteins

NADH and FADH_2_ generated from TCA cycle transfer electrons to the Fe-S cluster proteins in ETC complex I/II and start the electron transport in ETC I-IV (Tran et al., 2019; Hsu et al., 2019). Another important marker of cuproptosis is triggering the instability of Fe-S cluster proteins (Tsvetkov et al., 2022). The Fe-S cluster proteins expression can be determined by Western blot, IHC (indicated by Fe-S cluster protein antibody) and qRT-PCR assays. The assembly and transfer proteins of Fe-S cluster proteins involve: disulfurase complex NFS1-ISD11, frataxin, ferredoxin, ferredoxin reductase, mitochondrial carrier Mrs3/4, iron-sulfur cluster assembly 1/2 (Isa1/2), ironsulfur cluster assembly factor for biotin synthase and aconitase like mitochondrial proteins (57 kDa, Iba57), nucleoside hydrolase Ind1, assembly factor Nfu1, Fe-S protein subunits (NDUFS1, NDUFV1, SDHA, UQCRFS1), and so on (Sheftel et al., 2010; Terali et al., 2013; Schmucker et al., 2011; Moore et al., 2018; Sheftel et al., 2012; Muehlenhoff et al., 2011; Camponeschi et al., 2022; Da Vela et al., 2023; Chipuk, 2019; Ren L. et al., 2023; Maio et al., 2016; Sato et al., 2018). Herein, the expression of Fe-S cluster-related assembly and transfer proteins are significant for evaluating cuproptosis, and based on the instability of Fe-S cluster proteins, the detection results are suggested for negative trend.

### 5.3 HSP70

Heat shock proteins (HSPs), as molecular chaperons, play a protective role against the misfolding, aggregation and complex assembly disorder of cellular proteins under stress (Abd El-Fattah and Zakaria, 2022). HSP70, one of key HSPs, is low expressed and difficult to be detected in normal cells, but significantly upregulated under stress such as heat shock, oxidative stress, hypoxia and metal overload, which improves proteins misfolding, aggregation and sends them to ubiquitin-proteasome degradation or lysosome autophagy degradation pathways to avoid cell damage (Gráf et al., 2018). Importantly, during cuproptosis, HSP70 was the third marker and found to be upregulated in response to Cu overload and DLAT aggregation (Tsvetkov et al., 2022). Thus, the expression of HSP70 should be involved in cuproptosis verification using Western blot, IHC (indicated by HSP70 antibody) and qRT-PCR assays.

### 5.4 Cu transport and storage: ceruloplasmin, SLC31A1, ATP7A/7B, MT

**FIGURE 4:**
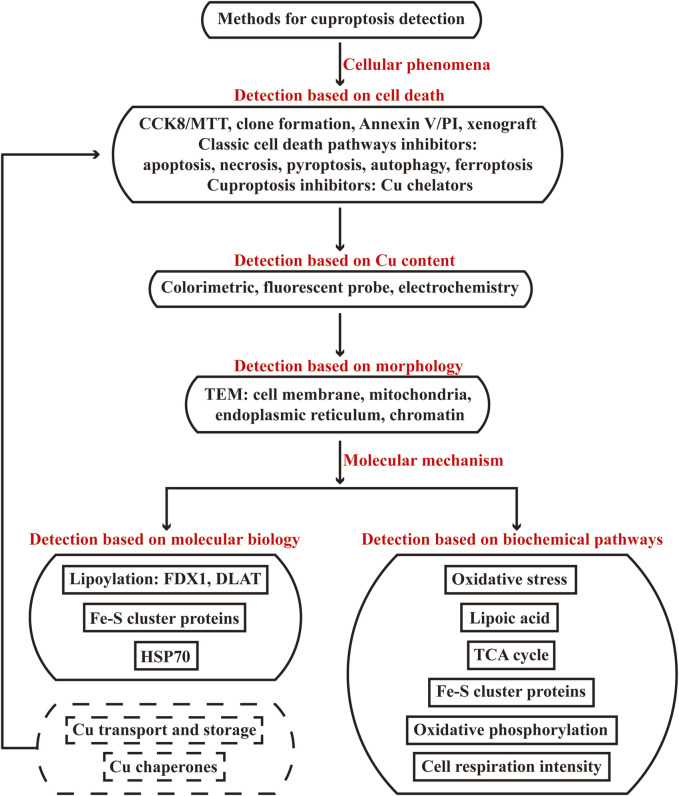
Flow chart of methods used to detect cuproptosis. The flow chart is categorized in two parts of cellular phenomena and molecular mechanism methods used to detect cuproptosis. The first part includes detection based on cell death, Cu content and morphology, the second part includes detection based on molecular biology of protein levels of cuproptosis-related molecules, biochemical pathways of cuproptosis-related enzyme activity and metabolites of oxidative stress, lipoic acid pathway, TCA cycle, Fe-S cluster proteins, oxidative phosphorylation, cell respiration intensity. The endogenous changes of protein levels of Cu transport and storage, Cu chaperones belong to molecular biology section, but such changes do not mean the induction of Cu cell death and need complete detection to verify the cell death effects and mechanisms existed through the flow chart.

Cu ions are absorbed from gastrointestinal tract and delivered to target cells by blood ceruloplasmin; SLC31A1, a membrane Cu importer, is internalized when there are sufficient intracellular Cu ions; ATP7A/B are present in Golgi apparatus network to pump in Cu ions for cuproenzyme metalation, but these proteins migrate to efflux Cu ions out of cells in Cu overload; the plasma protein metallothionein (MT) modulates the storage system of Cu ions, increasing expression level of MT suggests increasing metal levels and Cu toxicity in Cu stress (Nishito et al., 2024). Additionally, it has been revealed that it’s the mutation of ATP7B gene that results in Cu deposition in Wilson’s disease, accompanied with low levels of ceruloplasmin and Cu ions in plasma (Kirk et al., 2024; Bartee and Lutsenko, 2007). Lower expression level of ceruloplasmin in plasma suggests increasing level of Cu ions in hepatocytes, while higher expression level of MT suggests increasing intracellular Cu ions, which can be detected by Western blot, IHC (indicated by ceruloplasmin antibody and MT antibody) and qRT-PCR assays; IF assay can indicate the localization of SLC31A1 and ATP7A/B, the overexpression of SLC31A1 in membrane and low levels of ATP7A/B suggest more Cu import and less Cu efflux in cells; and the mutation of ATP7A/B measured by sequence alignment will also result in Cu deposition. Therefore, ceruloplasmin, SLC31A1, ATP7A/7B, MT are endogenous factors of Cu transport and storage to detect Cu toxicity. However, changing the expression status of ceruloplasmin, SLC31A1, ATP7A/7B, MT do not mean the induction of Cu cell death, these types of endogenous changes of Cu levels still need complete detection to verify the cell death effects and mechanisms existed through the flow chart of “Methods for cuproptosis detection” (Figure 4).

### 5.5 Cu chaperones: ATOX1, CCS, COX17

For intracellular Cu functions, Cu^+^ shuttle and activate the target enzymes through different protein carriers and mediate different biological functions (Figure 1). Antioxidant-1 (ATOX1) carries Cu^+^ to nucleus, where Cu^+^ bind to transcription complex and drive the expression of target genes associated with growth, metastasis, angiogenesis and so on (Blockhuys et al., 2020; Wang et al., 2023a). ATOX1 carries Cu^+^ to Golgi apparatus in which Cu^+^ are pumped in by ATP7A/B to activate enzyme activity of protein processing; when Cu ions become excess, ATP7A/B move from Golgi apparatus to export them out (Garza et al., 2023; Magistrato et al., 2019). Cu chaperone for superoxide dismutase (CCS) carries Cu^+^ to superoxide dismutase (SOD), where Cu^+^ participate in SOD eliminating free radicals for redox homeostasis (Luchinat and Banci, 2018). Cytochrome c oxidase 17 (COX17) carries Cu^+^ to synthesis of cytochrome c oxidase 1 (SCO1) and COX11 for cytochrome c oxidase (CCO) assembly in mitochondrion, where Cu^+^ activate enzyme activity of ETC (Zhu et al., 2023). The expression status of Cu chaperones indicate Cu utilization, which can be determined via Western blot, IHC (indicated by ATOX1 antibody, CCS antibody, COX17 antibody) and qRT-PCR assays. If ATOX1, CCS, COX17 decrease, it may suggests Cu accumulation. Thus ATOX1, CCS, COX17 are another endogenous factors of Cu chaperones to detect Cu overload. Similarly, changing the expression status of ATOX1, CCS, COX17 also do not mean the induction of Cu cell death, they need complete detection in response to the flow chart of “Methods for cuproptosis detection” (Figure 4).

## 6 Detection based on biochemical pathways

Cuproptosis initiates when excess Cu ions induce massive reactive oxygen species (ROS), bind to lipoylated DLAT, cause the aggregation of DLAT and the instability of Fe-S cluster proteins, which in turn disrupt the normal biochemical pathways of oxidative stress, lipoic acid, TCA cycle, Fe-S cluster proteins, oxidative phosphorylation, cell respiration intensity, and ultimately lead to cell death (Figure 2) (Tsvetkov et al., 2022). Herein, these biochemical pathways are vital indicators for evaluating the cell death effects in response to excess Cu ions. We focus on the enzyme activity (indicated by substrate consumption and product generation) and metabolites involved in these biochemical pathways, using ELISA, colorimetric, fluorescent, fluorometric, luminescent methods, respectively (Table 2).

**TABLE 2:** Common methods to detect biochemical pathways involved in cuproptosis

Targets	Assays	Principle	Ref.
Oxidative stress			
GSH	Abcam #ab138881	Fluorescent	Wang et al. (2022)
SOD	Abcam #ab65354	Colorimetric	Zhang et al. (2022b)
ROS	Abcam #ab219931 Abcam #ab113851	Fluorescent	Mursaleen et al. (2021) Scur et al. (2022)
Lipoic acid pathway			
SAM	Cloud-Clone #KSG414Ge11	Competitive ELISA	Shen et al. (2021)
PDH	Abcam #ab109902	Colorimetric	Yang et al. (2021)
KGDH	Abcam #ab185440	Colorimetric	Atlante et al. (2018)
Pyruvate	Elabscience #E-BC-K130-S	Colorimetric	Obaid et al. (2022)
α-ketoglutarate	Abcam #ab83431	Fluorometric	Wang et al. (2020b)
Acetyl-CoA	Abcam #ab87546	Fluorescent	Puca et al. (2021)
Succinate	Abcam #ab204718	Colorimetric	Stifel et al. (2022)
TCA cycle			
Citrate synthase	Abcam #ab119692	Colorimetric	Meng et al. (2021)
IDH	Abcam #ab102528	Colorimetric	Vaziri-Gohar et al. (2022)
KGDH	Abcam #ab185440	Colorimetric	Atlante et al. (2018)
NADH	Abcam #ab65348	Colorimetric	Ch et al. (2021)
FADH_2_	Fantaibio #FT-P36540R	Sandwich ELISA	—
Fe-S cluster proteins			
SAM	Cloud-Clone #KSG414Ge11	Competitive ELISA	Shen et al. (2021)
Aconitase	Abcam #ab109712	Colorimetric	Bailey et al. (2019)
PDH	Abcam #ab109902	Colorimetric	Yang et al. (2021)
KGDH	Abcam #ab185440	Colorimetric	Atlante et al. (2018)
Membrane potential	Elabscience #E-CK-A301	Fluorescent	Xiao et al. (2023)
Mitochondrial complex I	Elabscience #E-BC-K149-M	Colorimetric	—
Mitochondrial complex II	Elabscience #E-BC-K150-M	Colorimetric	—
Mitochondrial complex III	Elabscience #E-BC-K151-M	Colorimetric	—
Mitochondrial complex IV	Elabscience #E-BC-K152-M	Colorimetric	—
Oxidative phosphorylation			
Mitochondrial complex V	Abcam #ab109714	Colorimetric	Ge et al. (2021)
ATP	Abcam #ab113849	Luminescent	Ma et al. (2021)
Cell respiration intensity			
Oxygen consumption rate	Seahorse technology	—	Cinquegrani et al. (2022)
LDH	Abcam #ab102526	Colorimetric	Seki et al. (2022)

GSH, glutathione; IDH, isocitrate dehydrogenase; KGDH, α-ketoglutarate dehydrogenase; LDH, lactic dehydrogenase; PDH, pyruvate dehydrogenase; ROS, reactive oxygen species; SAM, S-adenosylmethionine; SOD, superoxide dismutase.

### 6.1 Oxidative stress: GSH, SOD, ROS

Glutathione (GSH) is one of the cellular Cu chelators and excess Cu^2+^ oxidize the reductive GSH into oxidized glutathione (GSSG), which makes exhaustion of GSH and impairs GSH antioxidant system (Li et al., 2022). Fenton reaction mediated by excess Cu^+^ catalyzes the synthesis of active hydroxyl radicals (·OH, OH^−^) (Jomova et al., 2022). Moreover, SOD with cofactor Cu^+^ eliminates free radicals and excess Cu^+^ will destroy the redox homeostasis (O_2_^−^) modulated by SOD (Magistrato et al., 2019). GSH/SOD antioxidant system failure and Cu Fenton reaction will result in increasing ROS levels (·OH, OH^−^, O_2_^−^) and cell toxic damage. Herein, in Cu stress, GSH level and SOD activity are decreased, accompanied with increasing ROS levels, fluorescent and colorimetric methods listed in “Oxidative stress” can indicate the quantitative results, respectively (Wang et al., 2022; Zhang W. et al., 2022; Mursaleen et al., 2021; Scur et al., 2022).

### 6.2 Lipoic acid: SAM, PDH, KGDH, pyruvate, α-ketoglutarate, acetyl-CoA, succinate

Lipoic acid is an eight-carbon saturated fatty acid that connects two sulfur atoms. It has strong antioxidant properties to directly remove ROS and can chelate heavy metal ions (Tsvetkov et al., 2022; Bera et al., 2023). Lipoic acid connects with target enzyme proteins through an amide bond and lipoylated modification after translation to maintain the catalytic activity, such as DLAT in PDH complex and dihydrolipoamide succinyltransferase (DLST) in α-ketoglutarate dehydrogenase (KGDH complex) (Koufaki et al., 2004; Cronan, 2016; Cronan, 2018). Lipoic acid transfers intermediate products between the active center of multi-enzyme complex, for example acyl group is transferred from lipoic acid to coenzyme A (CoA) (Cronan, 2020). FDX1 cooperated with LIAS are upstream regulators of protein lipoylation and their enzyme activities are impaired during cuproptosis, leading to the increase of S-adenosylmethionine (SAM, the substrate for LIAS), the inactivation of PDH complex and KGDH complex, the accumulation of pyruvate and α-ketoglutarate, the consumption of acetyl-CoA and succinate (Tsvetkov et al., 2022). Common methods to detect these enzyme activity, substrate levels and product levels are listed in “Lipoic acid pathway,” using ELISA, colorimetric, fluorometric and fluorescent methods, respectively (Shen et al., 2021; Yang et al., 2021; Atlante et al., 2018; Obaid et al., 2022; Wang Y. et al., 2020; Puca et al., 2021; Stifel et al., 2022).

### 6.3 TCA cycle: citrate synthase, IDH, KGDH, NADH, FADH_2_

In TCA cycle, pyruvate is converted to acetyl-CoA under the action of PDH complex, and acetyl-CoA plus oxaloacetate produce citrate, which initiates TCA cycle and subsequently generates isocitrate, cis-aconitate, oxalosuccinate, α-ketoglutarate, succinyl-CoA, succinate, fumarate, malate, oxaloacetate. Citrate synthase (Ren et al., 2020), isocitrate dehydrogenase (IDH) (Huang et al., 2019) and KGDH (Hansen and Gibson, 2022) catalyze the rate-limiting steps in TCA cycle. NADH and FADH_2_ are generated in TCA cycle and transported to ETC for electron transfer (Tran et al., 2019; Hsu et al., 2019). Cuproptosis induces the aggregation of lipoylated enzyme proteins (DLAT in PDH complex and DLST in KGDH complex) that involve in pyruvate converting to acetyl-CoA and the progress of TCA cycle, which inactivate the rate-limiting enzyme proteins of citrate synthase, IDH, KGDH and reduce the product NADH, FADH_2_ of TCA cycle. The enzyme protein activity and product quantitative methods are listed in “TCA cycle”, using colorimetric and ELISA methods, respectively (Meng et al., 2021; Vaziri-Gohar et al., 2022; Atlante et al., 2018; Ch et al., 2021).

### 6.4 Fe-S cluster proteins: prosthetic groups (LIAS, aconitase, mitochondrial complex I-III) and downstream targets (PDH, KGDH, mitochondrial complex IV)

Fe-S cluster proteins are prosthetic groups of LIAS (indicated by SAM consumption), aconitase, mitochondrial complex I-III, the instability of Fe-S cluster proteins during cuproptosis will decrease their enzyme activity, leading to low activity of LIAS-dependent PDH, LIAS-dependent KGDH and ETC (indicated by membrane potential and mitochondrial complex I-IV) (Tan et al., 2009; Navarro-Sastre et al., 2011; Tong et al., 2003; Cameron et al., 2011). Common methods to detect these enzyme activity of prosthetic groups and downstream targets in response to Fe-S cluster proteins instability are listed in “Fe-S cluster proteins,” using ELISA, colorimetric and fluorescent methods, respectively (Shen et al., 2021; Bailey et al., 2019; Yang et al., 2021; Atlante et al., 2018; Xiao et al., 2023).

### 6.5 Oxidative phosphorylation: mitochondrial complex V, ATP

H^+^ potential energy and free energy released by electrons transfer are used to produce ATP in mitochondrial complex V (Manoj et al., 2023). The reduction of TCA cycle and ETC activity induced by cuproptosis will decrease the enzyme activity of mitochondrial complex V and ATP production. Their detection methods are listed in “Oxidative phosphorylation,” using colorimetric and luminescent methods, respectively (Ge et al., 2021; Ma et al., 2021).

### 6.6 Cell respiration intensity: oxygen consumption rate, LDH

Cells highly dependent on mitochondrial respiration are much more sensitive to Cu toxicity than glycolytic cells, indicating that cuproptosis is mitochondrial respiration dependent (Tsvetkov et al., 2022). But the cell respiration may favor glycolysis from mitochondrial respiration due to excess Cu ions disrupting lipoylated TCA enzymes and ETC. Thus, the cell respiration intensity can be auxiliary indicators to evaluate the induction of cuproptosis. If cuproptosis predominates, the oxygen consumption rate will decrease from a high level and the enzyme activity of lactic dehydrogenase (LDH) will increase from a low level, which can be determined by Seahorse technology and LDH activity assay kit, respectively (“Cell respiration intensity”) (Cinquegrani et al., 2022; O'Day et al., 2013; Seki et al., 2022).

## 7 Comparison of cuproptosis and ferroptosis detection

In 2012, Stockwell BR et al. discovered a Fe-dependent form of nonapoptotic cell death named ferroptosis, characterizing by lipid peroxidation and excess ROS (Dixon et al., 2012). In our previous study, we demonstrated that the morpholine derivative N-(4-morpholinomethylene)ethanesulfonamide (MESA) could induce ferroptosis effect via targeting nuclear factor erythroid 2-related factor 2 (NRF2) signal pathways and suppress the viability of tumor cells (Sun et al., 2024). Thus the disorders of Fe and Cu ions metabolism have vital research value for the exploration of disease mechanisms and the development of therapeutic targets. In the past 10 years, ferroptosis has received great interests in the field of cell death and well-established detection methods have been continuously developed for verifying ferroptosis, which can also be categorized into cell death, Fe content, morphology, molecular biology and biochemical pathways according to the characteristic changes of ferroptosis. Techniques based on detecting the cell death effects are familiar with cuproptosis, accompanied with ferroptosis inhibitors; fluorescent probe (FerroOrange) is a common and specific technique for Fe content detection (Tian et al., 2021); morphological properties of ferroptosis include cell rounding cell membrane rupture, chromatin damage and more mitochondrial changes: small size, increased membrane density, outer membrane rupture, vanished crista, reduced membrane potential (Dixon et al., 2012); molecular biology can investigate the protein levels of ferroptosis-related molecules such as glutathione peroxidase 4 (GPX4) solute carrier family 7 member 12 (SLC7A12), acyl-CoA synthetase long-chain family member 4 (ACSL4), NRF2, Kelch-like ECH-associated protein 1 (KEAP1), P62 and so on (Wang L. et al., 2020); biochemical pathways of ferroptosis involve in GPX4, mevalonatepath, GSH, lipid peroxidation, oxidative stress and so on (Stockwell et al., 2017). Meanwhile, detection methods of cuproptosis are also summarized in Table 3 compared with that of ferroptosis, and they have some similarities of cell death effects, morphology, biochemical pathways (GSH, oxidative stress). Herein, we should focus our investigation on cell death specific inhibitors, ion content, cuproptosis-related molecules and cuproptosis specific biochemical pathways when verifying the occurrence of cuproptosis and distinguishing ferroptosis and other classic cell death pathways.

**TABLE 3:** Comparison of detection methods between cuproptosis and ferroptosis.

Type	Definition	Cell death	Ion content	Morphology	Molecular biology
Cuproptosis	Cu dependent: TCA cycle lipoylated proteins aggregation and Fe-S cluster proteins instability-mediated cell death	CCK8 MTT Clone formation Annexin V/PI Xenograft Inhibitors	Colorimetric Fluorescent probe Electrochemistry Photo electrochemistry	Cell membrane rupture Mitochondrial contraction Endoplasmic reticulum Chromatin damage	Ceruloplasmin, MT SLC31A1, ATP7A/7B ATOX1, CCS, COX17 FDX1, LIAS DLAT, Fe-S cluster HSP70
Ferroptosis	Fe dependent: lipid peroxidation and excess ROS-mediated cell death	CCK8 MTT Clone formation Annexin V/PI Xenograft Inhibitors	Fluorescent probe (FerroOrange)	Cell rounding Cell membrane rupture Chromatin damage Mitochondrial: small size, increased membrane density, outer membrane rupture, vanished crista, reduced membrane potential	GPX4, SLC7A12 ACSL4, NRF2 KEAP1, P62

ACSL4, acyl-CoA synthetase long-chain family member 4; ATOX1, antioxidant-1; CCS, Cu chaperone for superoxide dismutase; COX17, cytochrome c oxidase 17; DLAT, dihydrolipoamide S-acetyltransferase; FDX1, ferredoxin 1; GPX4, glutathione peroxidase 4; GSH, glutathione; HSP70,: heat shock protein 70; KEAP1, Kelch-like ECH-associated protein 1; LIAS, lipoyl synthase; MT, metallothionein; NRF2, nuclear factor erythroid 2-related factor 2; ROS, reactive oxygen species; SLC31A1, solute carrier family 31 member 1; TCA cycle, tricarboxylic acid cycle.

## 8 Discussion

Normal and stable Cu homeostasis plays an important role in a wide range of physiological process, while Cu overload will lead to cell damage and diseases of Cu toxicity. Cuproptosis is a newly discovered RCD in 2022 caused by excess Cu ions, which will induce cell damage due to aggregation of lipoylated DLAT, loss of Fe-S cluster proteins, upregulation of HSP70, proteotoxic and oxidative stress. Varying from other classic RCD, cuproptosis is highly concerned in many research fields nowadays, thereby effective detection methods to verify cuproptosis are an essential premise for cuproptosis studies. For this purpose, the principle and application of common cuproptosis detection methods are reviewed in this study according to the characteristic changes of Cu-dependent cell death, including cell death verification, Cu content, morphology, molecular biology of protein levels of cuproptosis-related molecules and biochemical pathways of cuproptosis-related enzyme activity and metabolites of oxidative stress, lipoic acid, TCA cycle, Fe-S cluster proteins, oxidative phosphorylation, cell respiration intensity. According to the markers of cuproptosis, we offer a flow chart of “Methods for cuproptosis detection” that categorizes into two part of cellular phenomena and molecular mechanism. The former including detection based on cell death, Cu content, morphology are employed to preliminarily verify the occurrence of cuproptosis, while the latter including detection based on molecular biology and biochemical pathways are employed to further gain comprehensive evaluation of cellular changes induced by cuproptosis with quantitative methods. All the indicating results have been displayed in the detection of cellular phenomena and molecular mechanism in response to the markers of cuproptosis, accompanied with the advantages and limitations.

Importantly, cuproptosis should be identified step by step in Cu stress. For cell death verification, the inhibitory effects on cell viability should not be sensitive to other classic cell death inhibitors (apoptosis, necrosis, autophagy, ferroptosis, etc.) but can be rescued by pretreatment of Cu chelators. For Cu content, ICP-MS is advanced for detecting Cu^2+^ and Cu^+^ but limited for application. We introduce three simple, sensitive, accurate and high-throughput methods of colorimetric, fluorescent probe and electrochemistry assays to effectively measure the Cu^2+^ content in serum, cell lysate and other samples. These three methods can be applied to readily detect Cu content according to the sensitivity for samples and the instrument for manipulation. For morphology, TEM can suggest the morphological features of cuproptosis with complicated sample preparation and subjective results of cell membrane rupture, mitochondrial contraction, endoplasmic reticulum and chromatin damage. Therefore, detection of cellular phenomena based on cell death effects sensitive to pretreatment of Cu chelators, Cu overload and specific morphological characteristics can only preliminarily indicate the induction of cuproptosis, further detection of molecular mechanism is needed to gain comprehensive evaluation of cellular changes induced by cuproptosis.

More quantitative detection methods are collected to completely evaluate the cellular changes in protein levels of cuproptosis-related molecules and in cuproptosis-related enzyme activity and metabolites in response to the markers of Cu-dependent cell death. For molecular biology, the characteristic changes of cuproptosis are abnormal aggregation of DLAT, loss of Fe-S cluster proteins and upregulation of HSP70, and the aggregation of DLAT involves the lipoylation regulated by DLAT/LIAS and the Cu binding to lipoic acid. The results of qRT-PCR assay in transcriptional level and Western blot, IHC assays can indicate the protein level changes of FDX1, DLAT, Fe-S cluster proteins, HSP70 in cells and tissues; the results of immunoprecipitation, mass spectrometry assays can indicate the lipoylation of DLAT; the aggregation of Cu^+^-DLAT can be captured by IF, nondenaturing gel electrophoresis/Western blot, HPLC, DLS, TDA, ES-DMA. Although the endogenous changes of protein levels of Cu transport and storage including increasing SLC31A1, MT, decreasing ceruloplasmin, decreasing/mutated ATP7A/7B and Cu chaperones including decreasing ATOX1, CCS, COX17 may lead to Cu accumulation, such changes do not mean the induction of cuproptosis and still need complete detection to verify the cell death effects and mechanisms existed through the flow chart we offer for cuproptosis detection (Figure 4). Similarly, the protein levels involved can be measured by qRT-PCR, Western blot, IHC assays, the localization of SLC31A1 and ATP7A/B can be measured by IF assay, and the mutation of ATP7A/B should be measured by sequence alignment. For biochemical pathways, cuproptosis has been revealed to disrupt the normal cellular metabolic pathways of oxidative stress, lipoic acid, TCA cycle, Fe-S cluster proteins, oxidative phosphorylation and cell respiration intensity (Figures 2, 4). Thus these biochemical pathways of cuproptosis-related enzyme activity and metabolites are vital indicators for cuproptosis verification and evaluation. We collect methods of ELISA, colorimetric, fluorescent, fluorometric and luminescent assays to measure the cuproptosis-related enzyme activity and metabolites in Cu stress, including GSH, SOD, ROS in oxidative stress, SAM, PDH, KGDH, pyruvate, α-ketoglutarate, acetyl-CoA, succinate in lipoic acid pathway, citrate synthase, IDH, KGDH, NADH, FADH_2_ in TCA cycle, LIAS, aconitase, mitochondrial complex I-III, PDH, KGDH, mitochondrial complex IV in Fe-S cluster protein pathway, mitochondrial complex V, ATP in oxidative phosphorylation and oxygen consumption rate, LDH in cell respiration intensity. Most of the cuproptosis-related enzyme activity are decreased, but the enzyme activity of LDH increases in Cu stress because the cell respiration favors glycolysis instead of oxidative phosphorylation due to the Cu toxicity on TCA cycle and ETC. Notably, the detection based on molecular biology of protein levels of cuproptosis-related molecules and biochemical pathways of cuproptosis-related enzyme activity and metabolites are employed to evaluate the characteristic changes of cuproptosis in molecular level, after the preliminary verification of cuproptosis based on cell death effects, Cu content and morphological features. But only detecting these changes of molecular biology and biochemical pathways can not suggest the occurrence of cuproptosis so that the type of cell death may be identified as cuproptosis followed by our flow chart of methods based on cellular phenomena and molecular mechanism detection (Figure 4).

The detection methods of cuproptosis we offer are mainly based on the characteristic changes of cell death caused by excess Cu ions, and the proper methods applied to verify the occurrence of cuproptosis depend on the reagents and instruments in laboratory. In addition, considering the prospective significance of cuproptosis and ferroptosis, the metabolic disorder of other metal ions also have specific research value for pathology exploration and therapeutic target development (Tsvetkov et al., 2022; Sun et al., 2024). Both cuproptosis and ferroptosis are involved in mitochondrial metabolism, other reported metal ions related to mitochondrial function are calcium, potassium, sodium, magnesium, zinc, and the reported metal elements involved in cell death are zinc (Du et al., 2021), manganese (Zhao et al., 2019) and calcium (Bai et al., 2022). However, the action mode of cell death of these metal ions are not clear enough compared with cuproptosis and ferroptosis. We will keep going through the progress of metal elements-mediated cell death and the principle and application of detection methods involved, hoping to provide technical support for cell death verification and exploration based on metal elements.

## 9 Conclusion

Collectively, the new type of cell death cuproptosis can be verified and evaluated based on the characteristic markers in terms of cell death, Cu content, morphology, molecular biology of protein levels of cuproptosis-related molecules, biochemical pathways of cuproptosis-related enzyme activity and metabolites, distinguishing from ferroptosis and other classic cell death. The cellular phenomena and molecular mechanism detection methods of cuproptosis we collect would contribute to verifying and exploring cuproptosis in future studies.
